# Eight orthopedic surgeons achieved moderate to excellent reliability measuring the preoperative posterior tilt angle in 50 Garden-I and Garden-II femoral neck fractures

**DOI:** 10.1186/s13018-017-0632-x

**Published:** 2017-09-19

**Authors:** Filip C. Dolatowski, Sigurd Erik Hoelsbrekken

**Affiliations:** 1 Institute of Clinical Medicine, University of Oslo, Oslo, Norway; 2 Department of Orthopedics, Akershus University Hospital, 1478 Lørenskog, Norway; 3 Department of Orthopedic and Rheumatic surgery, Kongsvinger Hospital, P.O. Box 901, 2226 Kongsvinger, Norway

**Keywords:** Femoral neck fracture, Garden-I and Garden-II, Posterior tilt, Reliability, Agreement, Minimal detectable change, Repeatability

## Abstract

**Background:**

Studies of elderly patients with Garden-I and Garden-II femoral neck fractures (FNFs) suggest that a preoperative posterior tilt of the femoral head of at least 20° increases the risk of fixation failure. A recently published treatment algorithm recommended hemiarthroplasty over internal fixation for elderly patients with Garden-I and Garden-II FNFs and a preoperative posterior tilt of at least 20°. However, the reliability of the method used to measure the posterior tilt has not been assessed according to recommended standards for reliability trials.

**Methods:**

Four orthopedic registrars and four consultants measured the posterior tilt angle in 50 preoperative lateral radiographs at two occasions six weeks apart. We estimated inter- and intrarater reliability by intraclass correlation coefficient (ICC). We also assessed repeatability by the repeatability coefficient (RC) and agreement by the minimal detectable change (MDC). Based on the suggested cutoff value of 20°, we reported the overall percentage and specific agreement for the choice of implant.

**Results:**

Inter- and intrarater reliability for all raters was excellent with an ICC (95% CI) of 0.77 (0.69–0.85) and 0.77 (0.67–0.86), respectively. The RC was 13.9 and the MDC 14.1. Specific agreement for choosing arthroplasty was 61.3 and 54.6% for the first and second test occasion, respectively.

**Conclusions:**

Eight orthopedic surgeons measured the posterior tilt in 50 Garden-I and Garden-II FNFs and achieved excellent inter- and intrarater reliability. However, variations in repeated measurements and variations in measurements made by different raters, as assessed by the RC and the MDC respectively, ranged from 13.9° to 14.1°. The variations in posterior tilt measurements should be taken into account when choosing the type of implant for elderly patients with Garden-I and Garden-II femoral neck fractures.

## Background

Elderly patients with Garden-I and Garden-II femoral neck fractures (FNFs) treated with internal fixation may suffer from higher rates of complications such as fixation failure, nonunion, and avascular necrosis of the femoral head than previously acknowledged [1–3]. Recent trials identified a subgroup of Garden-I and Garden-II FNFs that had an increased risk of fixation failure. Those were elderly patients who presented with a posterior tilt of the femoral head of at least 20° measured on the preoperative lateral radiograph [1–3]. Primary arthroplasty could thus be a better alternative for this subgroup of elderly patients [4, 5]. Two studies suggested that elderly patients with Garden-I and Garden-II FNFs with a posterior tilt of ≥ 20° could benefit from arthroplasty, whereas patients with a posterior tilt of < 20° may be treated with internal fixation [1, 3]. However, the findings of another retrospective study contradicted these results [6], and an explanation could be a possibly poor reliability of posterior tilt measurements. Therefore, we evaluated the inter- and intrarater reliability of posterior tilt measurements according to standards for good reliability studies [7].

## Methods

### Study design and population

This study was part of a retrospective cohort study of elderly patients with Garden-I and Garden-II FNFs treated with two cancellous screws at Akershus University Hospital, Norway, between 2005 and 2012. The authors evaluated anteroposterior radiographs of the pelvis and classified the fractures according to the simplified Garden classification [8]. To assure that radiographs were representative, we randomly selected 50 supine cross-table lateral view radiographs from a cohort of 322 patients with Garden-I and Garden-II femoral neck fractures using computer software. Patient data from the same cohort have recently been published [3]. All lateral view radiographs were used independently of their quality to reduce the risk of selection bias.

### Radiographic measurements

**Fig. 1:**
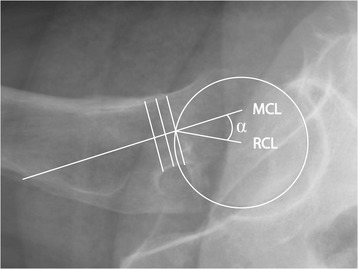
Garden-I and Garden-II femoral neck fracture—cross-table lateral view. The posterior tilt angle (α) is defined by the mid-collum line (MCL) and the radius collum line (RCL) [1]

The posterior tilt of the femoral head was measured with the software mDesk (RSA Medical, Umeå, Sweden) using the method described by Palm et al [1]. The raters fitted a circle to the cortical contour of the femoral head, and the software calculated the center point of the circle. The raters then drew a straight line across the narrowest part of the femoral neck succeeded by two parallel lines on each side, with a distance of 5 mm to the initial line. The mid-collum line (MCL) was defined as a line through the center points of the three lines. The radius collum line (RCL) was drawn from the center of the circle to the intersection between the circle and the MCL. The posterior tilt of the femoral head was defined as the angle between the MCL and RCL (Fig. 1).

Eight orthopedic surgeons—four registrars and four senior consultants—were invited to assess lateral hip radiographs at two occasions with a washout period of at least six weeks. The raters received individual instructions as described above for approximately 20 min before the first rating. None of the raters had any experience using the measuring method in question before the study. The raters were blinded to the clinical outcome and completed sessions independently at their pace, using the same portable computer and software. No feedback was provided between sessions, and the raters were not allowed to discuss their results. The inter- and intrarater reliabilities of measurements of posterior tilt were calculated based on the results of the first and second ratings.

### Statistics

Sample size calculations were performed according to the recommendations of Donner and Rotondi [9]. The eight raters were divided into two groups of four based on their clinical experience. For interrater analysis, intraclass correlation coefficients (ICCs) were estimated by a linear mixed model with random effects for patient and rater, which corresponds to a two-way mixed model, agreement and single measure (ICC 2.1). Calculations were performed using the R package *lme4* [10]. ICC was interpreted as follows [11]: excellent (> 0.75), fair to good (0.40–0.75), and poor (< 0.40). The standard error of measurement (SEM)_agreement_ was calculated from the square root of the sum of residual, patient, and rater variance. Minimal detectable change (MDC), which estimates the smallest amount of change that can be detected beyond measurement error, was calculated using the formula 1.96 × √2 × SEM.

The recorded posterior tilt angles were also dichotomized using the suggested cutoff value of 20° [1, 5] indicating the two implant options: arthroplasty ≥ 20° and internal fixation < 20°. The overall percentage agreement is the proportion of cases for which all raters agree, and the specific agreement was defined as the observed agreement for choosing arthroplasty as treatment. Percentage agreement was calculated with the R packages *obs.agree* [12].

Intrarater reliability (ICC_intra_) was estimated by a linear mixed model with random effects for patient, which corresponds to a two-way mixed model, agreement and single measure (ICC 2.1). The means of the individual ICC_intra_ with corresponding standard deviations (SDs) were used to compare intrarater reliability between groups of raters. Within-subject SD was calculated using one-way analysis of variance (ANOVA) and repeatability estimated by the repeatability coefficient (RC) using the formula √2 × 1.96 × within-subject SD [13]. Statistical analyses were performed using R version 3.1.3 for Mac OS X [14].

## Results

The eight raters measured posterior tilt in all 50 lateral hip radiographs at two test occasions, giving a total of 8 × 50 × 2 = 800 assessments (Appendix). The angles ranged from − 30.0 to 49.7°. Negative values denote anterior tilt of the femoral head, whereas positive values denote a posterior tilt. Using the mean angle of all eight measurements for each case from the first test occasion, 9 of 50 patients had a posterior tilt angle of at least 20°.

### Interrater reliability

The pair-wise ICC values for 28 possible pairs of raters ranged from “fair to good” (0.64) to “excellent” (0.91) (Table 1), and the overall ICC for the eight raters was “excellent” (0.77) at the first session (Table 2). The interrater reliability for registrars was “excellent” (0.81) compared to “fair to good” (0.73) for the consultants (Table 2), but the difference was not statistically significant (*p* = 0.19). Registrars achieved lower SEM and MDC values compared to the consultants (Table 2). Paired sample *t* test did not show any differences in reliability between the two test occasions (data not shown).

**Table 1:** Inter- and intrarater reliability for eight raters that evaluated the posterior tilt in 50 lateral radiographs of the hip

	R_1_	R_2_	R_3_	R_4_	C_1_	C_2_	C_3_	C_4_	ICC_intra_
R_1_	–	0.87	0.84	0.72	0.75	0.72	0.87	0.85	0.86
R_2_		–	0.91	0.78	0.82	0.71	0.86	0.91	0.90
R_3_			–	0.73	0.79	0.64	0.86	0.88	0.75
R_4_				–	0.78	0.64	0.70	0.81	0.82
C_1_					–	0.68	0.77	0.83	0.66
C_2_						–	0.70	0.68	0.62
C_3_							–	0.83	0.64
C_4_								–	0.89

*R*_*n*_ registrar N, *C*_*n*_ consultant N

**Table 2:** Interrater reliability for eight raters that evaluated the posterior tilt in 50 lateral radiographs of the hip

	ICC (95% CI)	SEM	MDC
8 raters	0.77 (0.69–0.85)	5.09	14.10
4 registrars	0.81 (0.72–0.87)	4.11	11.39
4 consultants	0.73 (0.65–0.84)	5.97	16.55

*CI*, confidence interval, *SEM* standard error of measurement, *MDC* minimal detectable change

### Intrarater reliability

Individual intrarater reliability (ICC_intra_) ranged from “fair to good” (0.62) to “excellent” (0.90) (Table 1, right column). The mean intrarater reliability for all raters was “excellent” (0.77) (Table 3). The mean ICC for the registrars was “excellent” (0.83) and for consultants “fair to good” (0.70), but the difference was not statistically significant (*p* = 0.12). Similar to SEM and MDC, the values for within-subject SD and RC were lower for registrars compared to the consultants (Table 3).

**Table 3:** Intrarater reliability for eight raters that evaluated the posterior tilt in 50 lateral radiographs of the hip

	Mean ICC (95% CI)	Within-subject SD	RC
8 raters	0.77 (0.67–0.86)	5.03	13.94
4 registrars	0.83 (0.73–0.93)	3.98	11.04
4 consultants	0.70 (0.50–0.90)	5.89	16.33

*CI* confidence interval, *SD* standard deviation, *RC* repeatability coefficient

### Agreement

The overall percentage agreement for all raters was 83.9 for the first test occasion and 82.1 for the second test occasion (Table 4). The specific agreement for choosing arthroplasty as treatment, based on the recommended cutoff value of a posterior tilt of at least 20°, was 61.3 and 54.6 for the first and second test occasions, respectively.

**Table 4:** Overall percent agreement (OPA) and specific agreement (SA) for arthroplasty when posterior tilt was ≥ 20°. Eight raters evaluated the posterior tilt in 50 lateral radiographs of the hip

	OPA (95% CI)	SA (95% CI)
8 raters	83.9 (78.4–89.2)	61.3 (41.3–75.2)
4 registrars	86.3 (80.0–92.3)	61.0 (34.8–78.0)
4 consultants	80.8 (74.4–87.2)	59.3 (41.5–73.4)

Numbers are percentages

## Discussion

Eight orthopedic surgeons measured the posterior tilt in 50 Garden-I and Garden-II FNFs and achieved excellent inter- and intrarater reliability. However, the MDC ranged from 11.4 to 16.6 and the RC from 13.9 to 16.3 (Tables 2 and 3).

We estimated inter -and intrarater reliability of posterior tilt measurements based on the ratings of four registrars and four consultants in orthopedic surgery. These measurements are of clinical importance because the presence of a preoperative posterior tilt in Garden-I and Garden-II FNFs has been associated with increased risk of fixation failure. In general, these fractures are treated with internal fixation, but arthroplasty has been recommended for fractures exceeding a cutoff value of 20° posterior tilt [1, 5]. To estimate variations in repeated measurements and variations in measurements made by different raters, we calculated the RC and the MDC. The RC represents the difference between two measurements made by the same rater on the same subject, and for 95% of pairs of observations, the difference will be less than the value of the RC. The MDC estimates the smallest change that can be detected beyond measurement error. We also evaluated the overall percentage agreement as well as specific agreement to provide information at a practical level.

ICC values for angular measurement were excellent, but the MDC was between 11.4 and 16.6 and the RC in the range of 13.9–16.3. These findings are relevant because variations in measurements of 15**°** are not inconsequential given the proposed treatment algorithm recommending internal fixation when the posterior tilt is < 20**°** and arthroplasty when the posterior tilt is ≥ 20**°** [5]. These observations could also partially explain discrepancies in the literature regarding the risk of treatment failure associated with preoperative posterior tilt [6].

Palm et al. reported excellent inter- and intrarater reliability among eight raters that evaluated posterior tilt in 17 Garden-I and Garden-II FNFs with ICC values of 0.87 (range 0.74–0.94) and 0.91 (range 0.83–0.95), respectively [5]. In the present study, the corresponding ICCs were also interpreted as excellent albeit with lower coefficients. Importantly, Palm et al. did not assess the repeatability or the MDC, but they did report inter- and intrarater kappa values for the choice of treatment and total percentage agreement for eight raters in 15 out of 17 cases (88.2%) [5]. In the present study, the total percentage agreement was between 82.1 and 83.9 for all raters. The specific agreement for choosing arthroplasty as treatment was 54.6–61.3%.

We recently reported excellent reliability of posterior tilt measurements performed by two orthopedic surgeons [3], with reliability similar to what has been published by Palm et al. In the present study, we invited eight orthopedic surgeons with no previous experience using the same measuring method and evaluated inter- and intrarater reliability. We used four registrars and four consultants to better reflect the staff of an orthopedic trauma hospital unit, and the resulting ICCs were lower than expected. The differences in reliability may also indicate that reliability could improve over time with more experience, although there was no improvement comparing the first and second measuring session.

We followed recommended guidelines for performing reliability studies [7]. The proportions of patients with a posterior tilt of at least 20° were similar in the randomly selected sample of 50 patients as compared to the cohort of 322 patients from which the sample was obtained [3]. The proportions of patients with a posterior tilt of at least 20° were 9 of 50 versus 43 of 322 (*p* = 0.38), and this supports the assumption that the sample of 50 patients was representative. Furthermore, there was no learning effect between the sessions as inter- and intrarater reliability was similar at both sessions, indicating that the second reading was independent of the first.

The present study has several limitations. None of the raters had any experience with the measuring procedure or the software used. Although we did not show any significant learning effect between the two rating occasions, a learning effect could still be present, accounting for higher reliability reported in previous studies. Furthermore, the software used in the present study differs from that used by Palm et al. in the original trial defining the measuring method [1]. The raters occasionally reported difficulties measuring posterior tilt due to poorly defined cortical contours. In a clinical setting, the clinician can acquire a new ﻿radiograph when image quality is poor, but we chose not to exclude lateral radiographs of poor quality to minimize the risk of selection bias. As a result, the reliability of posterior tilt measurements could be better in a clinical setting if radiographs of poor quality are replaced.

**Fig. 2:**
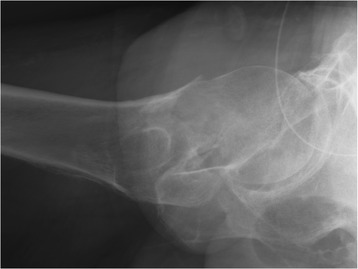
Garden-I and Garden-II femoral neck fracture—cross-table lateral view. Poorly defined cortical contours may cause the mid-collum line to deviate from the perceived central axis of the femoral neck

The mid-collum line could deviate substantially from the assumed central axis of the femoral neck, even though the three assisting lines were defined according to the procedure. These apparent mismatches occurred when the contours of the femoral neck were asymmetric or when radiographs demonstrated a double femoral neck contour (Fig. 2). As a result, the raters reported that they occasionally had to redefine the three assisting lines to achieve a reasonably oriented MCL.

## Conclusion

In the present retrospective cohort study, interpretations of inter- and intrarater reliability of posterior tilt measurements ranged from “fair to good” to “excellent.” The ICC values were lower than previously reported, and the MDC ranged from 11.4° to 16.6°. The specific agreement for choosing arthroplasty as treatment was 54.6–61.3%. The variations in posterior tilt measurements should be taken into account when choosing the type of implant for elderly patients with Garden-I and Garden-II femoral neck fractures.

## Appendix

**Table 5:** Four registrars and four consultants measured posterior tilt in 50 lateral hip radiographs at two test occasions

	Registrars								Consultants							
Case	R1t1	R1t2	R2t1	R2t2	R3t1	R3t2	R4t1	R4t2	C1t1	C1t2	C2t1	C2t2	C3t1	C3t2	C4t1	C4t2
1	5.4	2.7	1.8	0.3	3.7	− 4.5	7.3	− 3.5	1.1	1.6	2.6	− 0.1	3.3	8.4	− 2.5	0.3
2	12.6	16.1	20.6	18.8	17.9	21.3	19.9	20.1	18.4	17.9	16.5	19.9	17.5	18.8	16.9	21.6
3	15.3	13.5	17.9	21.6	25.0	11.4	6.5	4.4	12.2	14.7	14.4	10.6	18.3	21.6	20.5	13.7
4	1.6	12.4	16.9	12.0	18.4	7.9	23.7	8.3	16.6	5.9	14.6	14.2	8.6	14.9	17.0	8.3
5	11.9	14.9	11.2	9.8	5.7	6.0	2.6	13.3	2.0	4.9	7.7	13.8	11.5	13.4	11.0	10.9
6	1.0	1.6	− 4.5	− 1.3	4.4	11.0	5.4	1.1	1.4	14.7	18.3	5.4	− 3.0	3.5	0.2	2.9
7	1.2	2.8	− 0.8	− 1.0	2.7	2.8	1.3	− 2.5	0.1	0.7	0.6	0.0	0.2	1.3	− 3.0	0.0
8	− 1.0	− 5.1	− 1.9	− 5.4	0.9	0.2	0.2	0.6	− 15.9	14.4	− 5.4	8.6	− 4.9	16.0	− 5.3	8.9
9	11.0	20.5	20.9	19.9	10.7	19.4	25.5	16.7	22.5	20.9	27.2	15.6	22.4	21.4	22.7	20.2
10	7.6	7.6	5.2	8.5	0.9	9.5	8.0	8.5	6.6	11.4	20.3	10.5	0.6	8.3	11.3	5.5
11	15.1	9.6	10.7	14.2	11.9	14.8	21.9	18.2	10.0	20.1	24.6	21.2	15.4	12.9	18.2	12.6
12	19.0	11.6	24.2	21.7	19.2	21.4	19.8	19.7	16.4	18.1	22.4	16.3	23.5	15.3	17.0	22.2
13	− 13.8	− 17.9	− 11.6	− 15.3	− 10.5	0.2	− 9.7	− 9.6	− 3.6	1.5	− 14.1	9.1	− 17.9	9.9	− 6.4	− 7.9
14	0.2	1.9	10.2	7.8	3.5	11.5	8.1	5.7	0.7	7.5	10.6	4.8	2.9	11.8	8.0	7.1
15	8.4	17.6	13.0	10.6	8.3	24.7	8.3	16.3	16.5	10.1	18.8	8.7	7.7	18.1	8.3	10.1
16	7.6	3.9	8.2	3.8	7.8	− 8.2	32.2	21.3	11.7	13.2	12.6	8.1	5.2	9.1	13.2	7.0
17	28.6	28.7	19.9	34.8	20.2	19.3	23.0	22.0	21.8	26.0	46.3	18.4	32.5	31.2	19.0	22.8
18	17.6	16.1	13.5	17.3	14.3	15.6	11.3	16.9	11.7	15.9	19.0	17.9	14.8	15.0	16.5	14.8
19	10.8	12.7	17.3	17.1	16.3	13.2	13.2	20.5	17.2	18.1	26.0	11.8	13.7	16.1	18.3	17.6
20	34.2	41.9	32.7	32.0	31.3	36.5	29.8	34.0	28.3	42.7	49.7	32.7	35.0	27.3	27.5	26.7
21	1.7	0.1	0.5	2.4	1.9	− 1.8	1.7	2.9	10.4	7.0	3.1	− 8.8	15.8	4.0	2.6	− 2.1
22	23.0	16.2	21.2	21.3	25.0	21.1	17.0	13.7	22.8	16.5	40.5	23.1	23.2	25.0	23.3	21.6
23	11.4	23.7	15.2	16.4	18.8	13.8	23.1	31.5	32.9	13.7	22.0	14.1	12.5	23.4	13.8	11.5
24	23.0	24.6	27.2	25.0	23.7	19.1	25.0	30.8	23.9	25.1	47.1	23.7	22.3	30.1	18.5	20.5
25	26.3	28.0	22.2	27.9	23.5	28.2	21.6	18.6	29.0	26.0	38.3	26.3	25.9	26.2	27.7	24.3
26	25.3	24.0	28.1	32.9	30.9	23.9	28.6	28.3	22.8	23.0	22.6	20.5	31.5	26.0	31.1	25.8
27	14.5	14.5	15.7	19.5	17.5	17.3	18.0	25.6	22.4	12.8	24.3	16.6	17.8	16.9	20.0	21.9
28	9.7	19.8	14.9	18.1	16.9	17.0	13.6	13.3	14.5	21.1	16.3	21.0	14.0	19.6	9.2	10.0
29	5.0	12.3	7.3	7.1	8.3	8.7	4.8	5.4	8.6	7.8	13.3	7.0	7.4	8.9	5.7	7.6
30	9.7	7.6	13.3	13.3	16.4	15.1	12.3	10.3	14.4	13.3	20.2	11.8	17.4	7.8	9.2	17.0
31	13.8	11.3	17.2	20.2	14.9	13.1	13.5	13.2	18.1	7.8	13.6	14.8	14.1	19.8	17.0	15.9
32	4.0	− 1.5	4.9	− 3.0	1.3	1.0	1.2	− 2.2	0.0	2.0	5.8	− 0.4	− 4.0	17.3	− 3.4	2.1
33	15.3	21.5	18.7	14.9	16.1	19.1	13.7	8.8	15.1	20.4	29.4	15.3	22.7	18.5	15.7	21.4
34	2.0	3.7	− 3.8	− 0.3	− 2.5	1.5	4.8	− 2.7	13.9	1.8	4.0	24.7	5.0	9.7	− 2.6	− 6.1
35	− 15.9	− 15.5	− 8.1	− 10.0	− 0.6	− 1.7	− 10.2	− 2.9	− 7.1	− 19.0	− 30.0	− 16.6	− 4.1	− 14.7	− 9.0	− 10.9
36	7.1	3.1	8.7	6.4	9.1	9.4	14.3	3.1	10.9	7.8	17.9	11.9	8.3	8.7	12.4	4.7
37	16.2	17.4	19.1	2.2	18.0	19.7	6.9	21.5	18.2	18.1	22.7	18.2	17.6	16.2	19.7	21.8
38	10.8	5.6	4.8	8.8	11.4	3.1	8.0	4.9	3.4	5.8	12.7	6.1	12.4	4.1	4.2	5.8
39	7.8	7.6	6.8	8.0	8.4	8.7	6.3	4.1	5.7	2.3	6.5	9.3	6.0	1.1	5.0	8.0
40	18.6	25.2	20.4	19.9	17.5	18.0	14.1	18.0	17.3	16.0	30.2	18.6	17.7	24.0	20.5	16.6
41	5.7	3.9	1.3	2.7	7.6	8.8	7.2	4.7	1.3	10.9	5.2	4.8	7.7	9.6	7.0	− 2.1
42	2.7	5.0	2.8	− 2.0	0.8	3.3	1.2	3.3	0.3	2.5	5.7	3.1	0.8	1.3	3.3	0.7
43	10.6	0.4	7.1	7.2	0.1	7.1	11.7	12.9	3.3	14.1	7.4	1.5	9.6	1.7	4.4	1.3
44	10.4	21.4	8.7	12.6	8.9	29.8	9.0	10.9	9.4	18.7	9.6	21.1	7.2	7.8	12.6	8.3
45	12.3	20.6	14.8	15.4	14.4	16.4	21.9	17.0	13.4	15.2	12.9	13.3	20.7	42.0	16.2	17.5
46	6.7	− 3.1	3.6	4.8	3.8	4.6	2.4	1.5	8.0	3.3	9.6	2.6	5.2	6.9	1.8	2.5
47	9.1	11.0	4.1	5.0	10.8	8.4	3.2	8.1	3.3	7.5	8.9	10.1	22.0	8.1	6.7	8.4
48	16.9	19.7	27.2	16.8	22.3	17.6	11.3	16.6	16.3	19.5	31.1	16.4	24.6	19.9	17.5	14.5
49	13.4	17.7	12.2	10.5	12.3	16.7	7.9	11.4	14.8	14.3	14.0	7.1	12.5	14.0	13.1	9.4
50	13.3	12.3	12.5	9.5	10.3	10.4	14.0	12.7	13.3	14.4	30.2	10.3	9.3	14.5	12.3	11.6

Numbers are degrees. Positive values denote posterior and negative values anterior tilt of the femoral head

*R* registrar, *C* consultant, *t1* first, *t2* second test occasion

## Abbreviations

CI: Confidence interval
FNFs: Femoral neck fractures
ICC: Intraclass correlation coefficient
MCL: Mid-collum line
MDC: Minimal detectable change
OPA: Overall percent agreement
RC: Repeatability coefficient
RCL: Radius collum line
SA: Specific agreement
SD: Standard deviation
SEM: Standard error of measurement
